# Differential Expression of Subsets of Genes Related to HDL Metabolism and Atherogenesis in the Peripheral Blood in Coronary Artery Disease

**DOI:** 10.3390/cimb45080431

**Published:** 2023-08-16

**Authors:** Alexander D. Dergunov, Elena V. Nosova, Alexandra V. Rozhkova, Margarita A. Vinogradina, Veronika B. Baserova, Mikhail A. Popov, Svetlana A. Limborska, Liudmila V. Dergunova

**Affiliations:** 1 National Medical Research Center for Therapy and Preventive Medicine, Petroverigsky Street 10, Moscow 101990, Russia; veron1996@rambler.ru; 2 Laboratory of Human Molecular Genetics, National Research Center “Kurchatov Institute”, Kurchatov Sq. 2, Moscow 123182, Russia; el.nosova94@mail.ru (E.V.N.); avrojk@yandex.ru (A.V.R.); vin-rita@yandex.ru (M.A.V.); limbor.img@yandex.ru (S.A.L.); dergunova-lv.img@yandex.ru (L.V.D.); 3 Moscow Regional Research and Clinical Institute MONIKI, Moscow 129110, Russia; popovcardio88@mail.ru

**Keywords:** atherosclerosis, coronary artery disease, differentially expressed genes, HDL metabolism, inflammation, transcript

## Abstract

Differential expression of genes (DEGs) in coronary artery disease (CAD) and the association between transcript level and high-density lipoprotein cholesterol (HDL-C) were studied with 76 male patients with CAD and 63 control patients. The transcript level of genes related to HDL metabolism (24 genes) and atherosclerosis-prone (41 genes) in RNA isolated from peripheral blood mononuclear cells was measured by real-time RT-PCR. Twenty-eight DEGs were identified. The expression of cholesterol transporters, *ALB*, *APOA1*, and *LCAT* was down-regulated, while the expression of *AMN*, *APOE*, *LDLR*, *LPL*, *PLTP*, *PRKACA*, and *CETP* was up-regulated. The systemic inflammation in CAD is evidenced by the up-regulation of *IL1B*, *TLR8, CXCL5*, and *TNFRSF1A*. For the controls, *TLR8* and *SOAT1* were negative predictors of the HDL-C level. For CAD patients, *PRKACG*, *PRKCQ*, and *SREBF1* were positive predictors, while *PRKACB*, *LCAT*, and *S100A8* were negative predictors. For CAD patients, the efficiency of reverse cholesterol transport is 73–79%, and intracellular free cholesterol seems to accumulate at hyperalphalipoproteinemia. Both atheroprotective (via *S100A8*) and proatherogenic (via *SREBF1*, *LCAT*, *PRKACG*, *PRKACB*, and *PRKCQ*) associations of gene expression with HDL-C determine HDL functionality in CAD patients. The selected key genes and involved pathways may represent HDL-specific targets for the diagnosis and treatment of CAD and atherosclerosis.

## 1. Introduction

Coronary atherosclerosis and coronary heart disease (CAD) are characterized by disturbances of lipoprotein metabolism, innate and adaptive immunity, and inflammation. Myeloid cells acquire proinflammatory features that activate the cascade of signaling events associated with cholesteryl ester accumulation in macrophages [1,2]. Foam cell formation is influenced in part by the proatherogenic and antiatherogenic balances of LDL and HDL, respectively. The cholesterol stationary level in macrophages is maintained by tight control of cholesterol influx with LDL and cholesterol efflux to HDL via cholesterol transporters [3] as a first step in reverse cholesterol transport to the liver for cholesterol excretion. The cholesterol efflux capacity (CEC) of HDL determines its antiatherogenic effect rather than HDL cholesterol level [4]. However, recent findings have challenged the casual relationship between CEC and atherosclerosis [5,6,7], and this issue is actively debated [1].

ABCA1-mediated cholesterol efflux is an exclusive route to lipid-free apoA-I, whereas ABCG1 and scavenger receptor BI (SR-BI) are involved in cholesterol efflux to HDL [8,9,10]. The second step includes the synthesis of cholesteryl ester (CE) molecules, which is catalyzed by lecithin:cholesterol acyltransferase (LCAT). The inclusion of hydrophobic CE molecules into the particle core results in the transition from discoidal to spherical shapes of HDL particles [11]. The third step includes the selective uptake of CE molecules from HDL particles by hepatocytes via SR-BI [12] (direct RCT) or the exchange of CE in HDL for triglycerides in LDL via the CE transfer protein (CETP). CE-enriched LDL particles bind to the LDL receptor in hepatocyte membranes and are internalized (indirect RCT). Modified LDL decreases the expression of SR-BI but induces the expression of SR-B [13]. The activity of the phospholipid-transfer protein (PLTP) induces the remodeling of large HDL particles with concomitant fusion–fission of these particles, which results in the regeneration of small HDL particles and the dissociation of lipid-free apoA-I [14].

The synthesis and uptake of triglyceride and cholesterol molecules are regulated by the sterol-regulatory element-binding proteins *SREBP1* and *SREBP2*, respectively. *SREBP1* increases fatty acid synthesis. Transcription of the *SREBP1c* gene is increased by insulin (an activator) and decreased by glucagon (an inhibitor) [15]. Transcription of the *SREBP1c* gene is also activated by liver X receptors (LXRs), thereby assuring a supply of fatty acids at sterol overloading to allow storage of excess cholesterol as cholesteryl ester [16]. LXRα (NR1H3) and LXRβ (NR1H2) are members of the nuclear hormone receptor superfamily of ligand-dependent transcription factors that regulate transcription in response to the direct binding of cholesterol derivatives [17]. *SREBP2* acts through the increase of transcription of target genes for cholesterol synthesis or uptake through the LDL receptor at the decrease of cholesterol levels in the ER membrane. SREBP1a in mouse macrophages links lipid metabolism to the innate immune response [18,19]. However, inconsistent data exist on *SREBP’s* expression at CAD [20,21]. Another cholesterol sensor, HMG CoA reductase, a rate-limiting step in cholesterol synthesis, is rapidly degraded at cholesterol accumulation in ER membranes [22]. The cholesterol in macrophages is esterified by acyl coenzyme A:cholesterol acyltransferase 1 (SOAT1 aka ACAT1) and is regenerated from cholesteryl ester by neutral cholesteryl ester hydrolase [23]. Cholesterol influx through the LDL receptor and scavenger receptor CD36 for oxidized LDL, cholesterol efflux, cholesterol synthesis, and storage as cholesteryl ester determine the cholesterol pool level in the plasma membrane accessible for efflux [24].

The existence of various cell populations in atheroma is rapidly recognized based mainly on single-cell RNA sequencing [25]. It has been assumed that atherosclerotic arteries contain several macrophage subsets endowed with specific functions [26]. Also, foamy and non-foamy macrophages have been suggested to exist in atherosclerotic plaque and lipid-loaded plaque macrophages are not likely the plaque macrophages that drive lesional inflammation [27], thus contrasting with the promotion of macrophage foam cell formation, activation of the NLRP3 inflammasome, and atherogenesis at *Abca1*/*Abcg1* deficiency [28]. The non-coincident changes in pro-inflammatory gene expression in foam cells compared to non-foam cells may complicate the overall analysis of lesional inflammation. Moreover, HDL may possess both anti- and pro-inflammatory effects via cholesterol efflux [29] and Toll-like receptor-induced PKC signaling [30], respectively. Thus, it seems reasonable to perform a combined study of the expression of genes involved in lipid metabolism and inflammation in relation to HDL levels in CAD.

Based on the available data on differential expression genes at CAD established by RNA-seq and the analysis of genes involved in lipoprotein metabolism and atherogenesis, we recently suggested two pools of genes (HDL cluster and atherogen cluster, 64 genes total) to perform an analysis of the expression of these genes with a conventional real-time RT-PCR [31]. In the present study, the *SOAT1* gene was added to the HDL cluster to analyze the traffic of intracellular cholesterol. We used two gene pools: first, to reveal the differential expression of genes in the HDL cluster and atherogen cluster in peripheral blood mononuclear cells from CAD patients; second, to relate gene expression to the metabolic pathways involved in CAD; finally, to establish the significance of particular transcripts with proatherogenic or atheroprotective properties as predictors of HDL level both in CAD and control patients in relation to HDL functionality. We collected blood samples from control and CAD patients unaffected by any previous lipid-lowering treatment to avoid any changes in lipid metabolism, immune status, or inflammation status induced by such treatment.

## 2. Materials and Methods

### 2.1. Patients and Laboratory Tests

One hundred thirty-nine Caucasian male subjects from Moscow and Moscow District, 40–60 years old, were divided into two cohorts. The first cohort (*n* = 63) included patients without coronary atherosclerosis, while the second one (*n* = 76) included patients with CAD and stenosis of coronary arteries verified by standard coronary angiography for each patient in the two cohorts. The hemodynamically significant stenosis was diagnosed in a case of more than 50% luminal narrowing in at least one vessel, and these patients were included in the CAD cohort. The patients without any visible stenosis or with hemodynamically insignificant (<20%) stenosis were included in the control cohort. All patients in the two cohorts were without hypertension or diabetes, not alcoholics, and not on treatment with corticosteroids or lipid-lowering drugs for at least 3 months before drawing a blood sample. The latter requirement originates from the known prominent changes in lipid metabolism induced by lipid-lowering therapy. To deal with unaffected blood samples, we searched for new patients without any previous hypolipidemic treatment, first by selecting patients with chest pain and with electrocardiogram and/or echocardiogram abnormalities at the stage of outpatient care, followed by coronary angiography to diagnose CAD, second by blood drawing, and, finally, by the beginning of adequate therapy, including lipid-lowering drugs. Written informed consent was obtained from each patient included in the study. The study protocol conforms to the ethical guidelines of the 1975 Declaration of Helsinki and has been approved by the local ethics committee for research on humans. The lipid profiles were determined enzymatically with commercial kits on an Architect c8000 autoanalyzer (Abbott, Abbott Park, IL, USA). Plasma apoA-I and apoB levels were measured with immunoturbidimetric kits from DiaSys (Holzheim, Germany) on a Sapphire 400 autoanalyzer (Hirose Electronic System, Tokyo, Japan).

### 2.2. Gene Expression Analysis by Real-Time RT-PCR

Ficoll–Hypaque (1.077 g/mL) density gradient centrifugation (Sigma, St. Louis, MO, USA) was performed to isolate peripheral blood mononuclear cells. Total RNA was isolated with TRI Reagent (Molecular Research Center, Cincinnati, OH, USA) and traces of DNA were removed by DNase I in the presence of an RNase inhibitor according to the manufacturer’s protocol (ThermoFisher Scientific, Waltham, MA, USA). RNA samples were kept at −70 °C. RNA concentration and quality (RQI > 9) were measured with the Experion electrophoresis system (Bio-Rad, Hercules, CA, USA). cDNA was synthesized with the RevertAid First Strand cDNA Synthesis Kit (ThermoFisher Scientific, Waltham, MA, USA), and cDNA samples were kept at −20 °C.

The HDL cluster included the following twenty-four genes (Supplementary Table S1): Albumin (*ALB*); Alpha-2-macroglobulin (*A2M*); Amnion associated transmembrane protein (*AMN*); Apolipoprotein A1 (*APOA1*); Apolipoprotein E (*APOE*); ATP binding cassette subfamily A member 1 (*ABCA1*); ATP binding cassette subfamily A member 5 (*ABCA5*); ATP binding cassette subfamily G member 1 (*ABCG1*); Bone morphogenetic protein 1 (*BMP1*); Cholesteryl ester transfer protein (*CETP*); Cubilin (*CUBN*); High density lipoprotein binding protein (*HDLBP*); 3-hydroxy-3-methylglutaryl-CoA reductase (*HMGCR*); Lecithin-cholesterol acyltransferase (*LCAT*); Lipase C, hepatic type (*LIPC*); Lipoprotein lipase (*LPL*); Low density lipoprotein receptor (*LDLR*); Phospholipid transfer protein (*PLTP*); Protein kinase cAMP-activated catalytic subunit alpha (*PRKACA*); Protein kinase cAMP-activated catalytic subunit beta (*PRKACB*); Protein kinase cAMP-activated catalytic subunit gamma (*PRKACG*); Scavenger receptor class B member 1 (*SCARB1*); Sterol O-acyltransferase 1 (*SOAT1*); Zinc finger DHHC-type containing 8 (*ZDHHC8*).

The atherogen cluster included the following 41 genes (Supplementary Table S1): Asialoglycoprotein receptor 2 (*ASGR2*); CD14 molecule (*CD14*); CD36 molecule (*CD36*); Coagulation factor V (*F5*); Colony stimulating factor 1 receptor (*CSF1R*); Colony stimulating factor 2 receptor beta common subunit (*CSF2RB*); C-X-C motif chemokine ligand 5 (*CXCL5*); Cytochrome b-245 alpha chain (*CYBA*); Integrin subunit alpha 2b (*ITGA2B*); Integrin subunit alpha M (*ITGAM*); Integrin subunit beta 3 (*ITGB3*); Intercellular adhesion molecule 1 (*ICAM1*); Interleukin 1 beta (*IL1B*); Interleukin 1 receptor type 1 (*IL1R1*); Interleukin 18 (*IL18*); Interleukin 18 receptor 1 (*IL18R1*); Interleukin 18 receptor accessory protein (*IL18RAP*); Junctional adhesion molecule 3 (*JAM3*); Lymphotoxin alpha (*LTA*); Matrix metallopeptidase 9 (*MMP9*); Microsomal glutathione S-transferase 1 (*MGST1*); NPC intracellular cholesterol transporter 1 (*NPC1*); NPC intracellular cholesterol transporter 2 (*NPC2*); Nuclear receptor subfamily 1 group H member 2 (*NR1H2*); Nuclear receptor subfamily 1 group H member 3 (*NR1H3*); Oxidized low density lipoprotein receptor 1 (*OLR1*); Phosphatidylcholine transfer protein (*PCTP*); Phospholipase A2 group VII (*PLA2G7*); Protein kinase C theta (*PRKCQ*); S100 calcium binding protein A12 (*S100A12*); S100 calcium binding protein A8 (*S100A8*); S100 calcium binding protein A9 (*S100A9*); Secretory leukocyte peptidase inhibitor (*SLPI*); Solute carrier family 7 member 11 (*SLC7A11*); Sterol regulatory element binding transcription factor 1 (*SREBF1*); Superoxide dismutase 2 (*SOD2*); TNF receptor superfamily member 1A (*TNFRSF1A*); TNF receptor superfamily member 1B (*TNFRSF1B*); Toll like receptor 5 (*TLR5*); Toll like receptor 8 (*TLR8*); Vascular endothelial growth factor A (*VEGFA*).

Housekeeping genes included Glyceraldehyde-3-phosphate dehydrogenase (*GAPDH*), Lactate dehydrogenase A (*LDHA*), and Ribosomal protein L3 (*RPL3*) genes (Supplementary Table S1).

Gene-specific primers were designed using OLIGO Primer Analysis Software 6.31 (Molecular Biology Insights, Colorado Springs, CO, USA). Primer structures and coordinates are given in Supplementary Table S1. The specificity of the primers was verified by the melting curve of the PCR product (a single peak). Real-time RT-PCR (RT-PCR) was carried out on a StepOnePlus Real-Time PCR System (Applied Biosystems, Foster City, CA, USA) in 25 µL of reaction mixture containing 2 µL of 1:30 or 1:6 diluted cDNA, 5 pmol each of forward and reverse primers, and 5 µL 5x PCRmix-HS SYBR (Evrogen, Moscow, Russia). The amplification protocol included: (1) a hot start at 95 °C for 10 min; (2) 40 cycles at 95 °C for 15 s, 65 °C for 25 s, and 72 °C for 35 s; data were collected at the elongation step; (3) melting curve was obtained by heating from 65 °C to 95 °C with a 0.3 °C step.

### 2.3. Interaction between Protein Products of Genes and Pathway Analyses

The differentially expressed genes were annotated by functional enrichment analysis with the Gene Ontology (GO) [32,33] and the Kyoto Encyclopedia of Genes and Genomes (KEGG) [34] pathway databases. The online tool Database for Annotation, Visualization, and Integrated Discovery (DAVID) [35] was explored to detect GO categories. The analysis of protein–protein interactions (PPI) was performed with the Search Tool for the Retrieval of Interacting Genes (STRING) database [36]. The data generated by the STRING database were visualized with Cytoscape [37] and KEGG pathway analysis was conducted with the Cytoscape functional enrichment module. The *p*-value for PPI enrichment is an indicator of interaction efficiency. The number of nodes and the number of edges are network characteristics.

### 2.4. Statistical Analysis

Statistica (version 13) software was used. The data are given as mean values with standard deviations. The Kolmogorov–Smirnov test was used to check the normality, and the variables with a skewed distribution were log-transformed. Differences in continuous variables between groups were analyzed by ANOVA with posteriori estimates by Fisher’s LSD test or the Mann–Whitney test. The statistical significance limit was accepted as *p* < 0.05. Pearson’s correlation coefficients were calculated to describe relationships between variables. Multiple linear regression was performed as well. The cohort sizes were sufficient for the robust correlation and regression analyses at the *p* = 0.05 level, as verified by power and sample size analyses. In multiple regression, the assumption that the residuals (predicted minus observed values) are distributed normally was verified by a normal probability plot of residuals. Furthermore, the Darbin–Watson statistic DW was used as a *test* for checking autocorrelation in the residuals; no first-order autocorrelation was assumed for DW values 1.50–2.50. The multicollinearity of the predictors in the regression equation was checked by the value of the variance inflation factor (VIF), which is a reciprocal of tolerance. Tolerance for the *i*th independent variable is 1 minus, the proportion of variance it shares with the other independent variable in the analysis; this represents the proportion of variance in the *i*th independent variable that is not related to the other independent variables in the model. The VIF value of 1 corresponds to the complete absence of multicollinearity, and the generally accepted VIF values lower than 4 correspond to negligible multicollinearity and the accurate values of regression coefficients [38]. Paired analysis of RT-PCR data was conducted with the REST-2009 software [39], and the data are given as a mean value of expression ratio with the standard error as a 68% confidence interval. The permutation test used in the REST-2009 software is designed to determine whether the observed difference between the sample means is large enough to reject; at some significance level, the null hypothesis that the data drawn from the control is from the same distribution as the data drawn from the treatment group. The data verified by the permutation test are much more robust and do not require any previous knowledge of the nature of the distribution. In addition, the relative expression of all target genes normalized by the geometric mean of three reference genes [40] was used in the analysis. The false discovery rate (FDR) for multiple comparisons was controlled by the Benjamini–Hochberg procedure [41]. The FDR value was fixed at the 0.05 level unless indicated. The raw *p*-values significant under the Benjamini–Hochberg procedure are given in the analysis of gene expression.

## 3. Results

### 3.1. Lipid and Lipoprotein Levels and Anthropometric Data of the Patients

The number of patients in the control and CAD cohorts was equal to 63 and 76 patients, respectively. Age, body mass index (BMI), plasma lipoprotein, and apolipoprotein data for patients in two cohorts are included in Table 1. The mean values of total cholesterol, LDL-C, nonHDL-C, and atherogenicity index were lower in CAD compared to controls, while apoA-I and age were significantly higher. The cohorts did not differ from each other by HDL-C, VLDL-C, plasma TG, apoB, or BMI. Notably, cholesterol content in a single LDL particle, calculated from the mean levels of Chol, LDL-C, TG, and apoB, was 25% lower for CAD patients compared to controls.

### 3.2. Expression of Genes Related to HDL Metabolism and Atherogenesis in Control and CAD Cohorts

#### 3.2.1. Differential Gene Expression

The transcript levels of genes related to HDL metabolism (HDL cluster) and atherogenesis (atherogen cluster) selected by us earlier [31,42] were measured by real-time RT-PCR. The *SOAT1* gene was additionally included in the HDL cluster to follow the intracellular cholesterol traffic. The differential expression at the transcript level in CAD versus control cohorts (fold change) was analyzed with the REST-2009 software. The multiple comparisons were controlled with the Benjamini–Hochberg procedure with an FDR value of 0.05.

**Figure 1:**
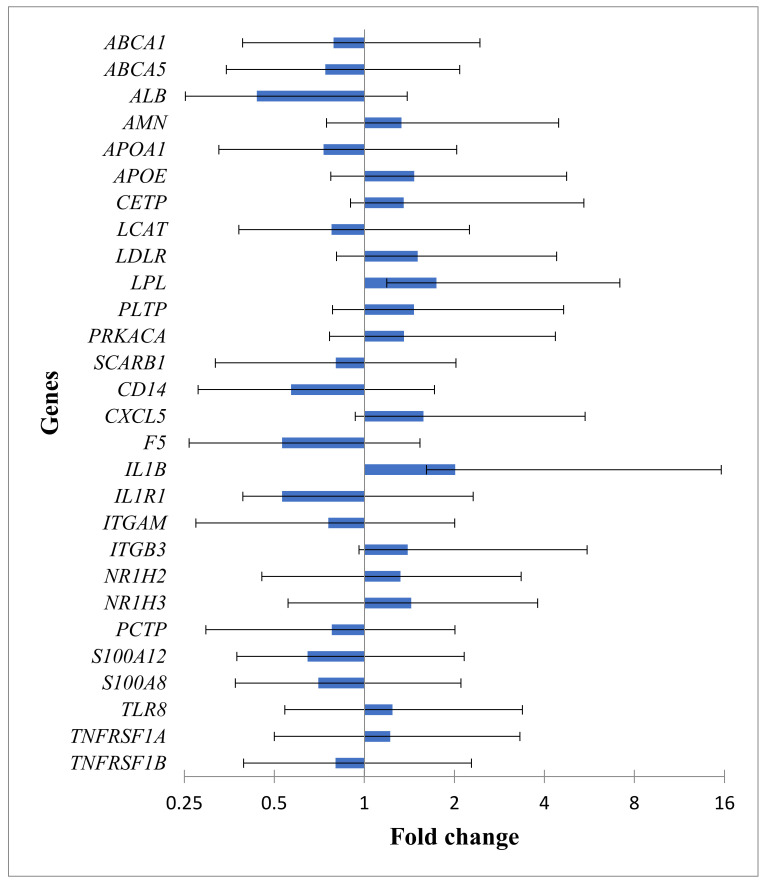
The change of gene expression in two gene clusters in CAD. The fold change for HDL and atherogen clusters was calculated with the REST-2009 software. Only significant changes controlled for multiple comparisons by the Benjamini–Hochberg procedure (FDR = 0.05) are given as mean (SE) values.

A total of 28 DEGs in CAD were identified by the REST, and the data are given in Figure 1. Compared with control, seven genes in the HDL cluster—amnion-associated transmembrane protein (*AMN*), apolipoprotein E (*APOE*), cholesteryl ester transfer protein (*CETP*), low density lipoprotein receptor (*LDLR*), lipoprotein lipase (*LPL*), phospholipid transfer protein (*PLTP*), and protein kinase cAMP-activated catalytic subunit alpha (*PRKACA*)—were up-regulated, and six genes, namely, ATP binding cassette subfamily A member 1 (*ABCA1*), ATP binding cassette subfamily A member 5 (*ABCA5*), albumin (*ALB*), apolipoprotein A1 (*APOA1*), lecithin-cholesterol acyltransferase (*LCAT*), and scavenger receptor class B member 1 (*SCARB1*), were down-regulated in CAD.

Analogously, seven genes in the atherogen cluster—C-X-C motif chemokine ligand 5 (*CXCL5*), interleukin 1 beta (*IL1B*), integrin subunit beta 3 (*ITGB3*), nuclear receptor subfamily 1 group H member 2 (*NR1H2*), nuclear receptor subfamily 1 group H member 3 (*NR1H3*), Toll-like receptor 8 (*TLR8*), and TNF receptor superfamily member 1A (*TNFRSF1A*)—were up-regulated, and eight genes—CD14 molecule (*CD14*), coagulation factor V (*F5*), interleukin 1 receptor type 1 (*IL1R1*), integrin subunit alpha M (*ITGAM*), phosphatidylcholine transfer protein (*PCTP*), S100 calcium-binding protein A12 (*S100A12*), S100 calcium-binding protein A8 *(S100A8*), and TNF receptor superfamily member 1B (*TNFRSF1B*)—were down-regulated.

#### 3.2.2. Functional Enrichment Analysis and Protein–Protein Interactions

Gene Ontology functional enrichment for 28 DEGs was analyzed with DAVID. The data sorted by FDR value is included in Table 2. The top 5 of 46 biological processes included GO:0042632~cholesterol homeostasis; GO:0034375~high-density lipoprotein particle remodeling; GO:0043691~reverse cholesterol transport; GO:0010745~negative regulation of macrophage-derived foam cell differentiation; GO:0006869~lipid transport. The top 5 of 12 molecular functions included GO:0031210~phosphatidylcholine binding; GO:0001540~beta-amyloid binding; GO:0008289~lipid binding; GO:0034186~apolipoprotein A-I binding; GO:0071813~lipoprotein particle binding. The top 5 of 17 cellular components included GO:0005615~extracellular space, GO:0005576~extracellular region, GO:0034364~high-density lipoprotein particle, GO:0005886~plasma membrane, and GO:0009897~external side of plasma membrane (Table 2).

**Figure 2:**
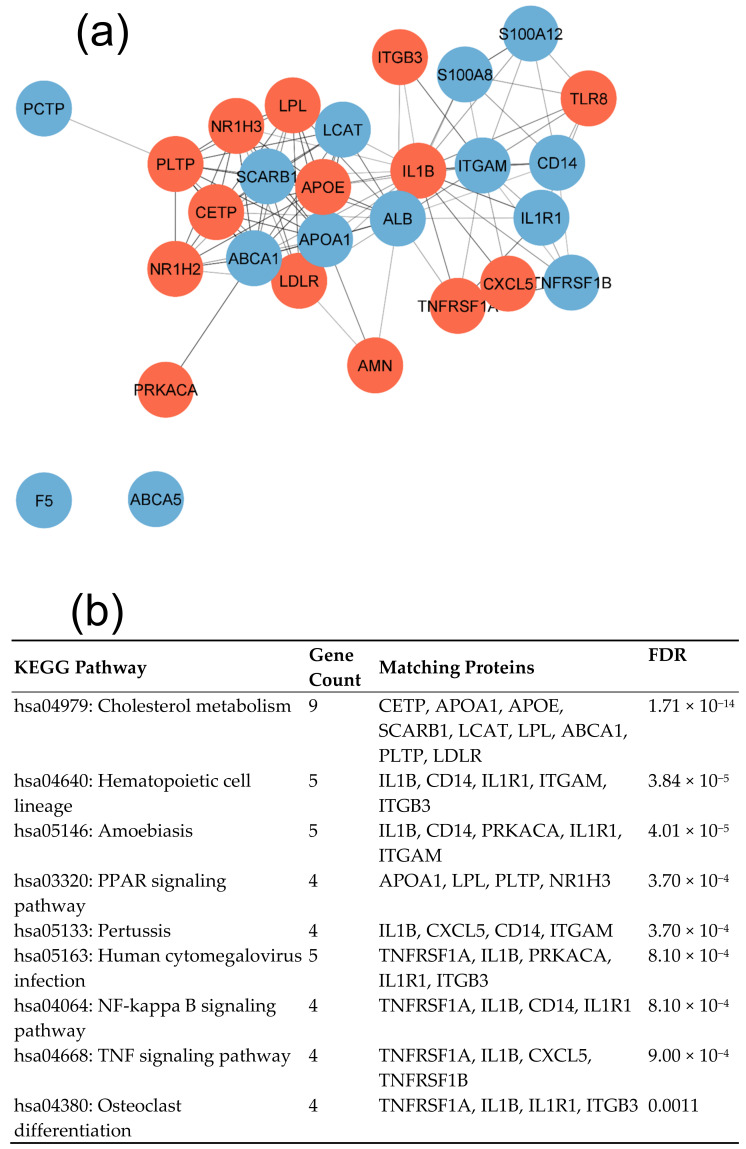

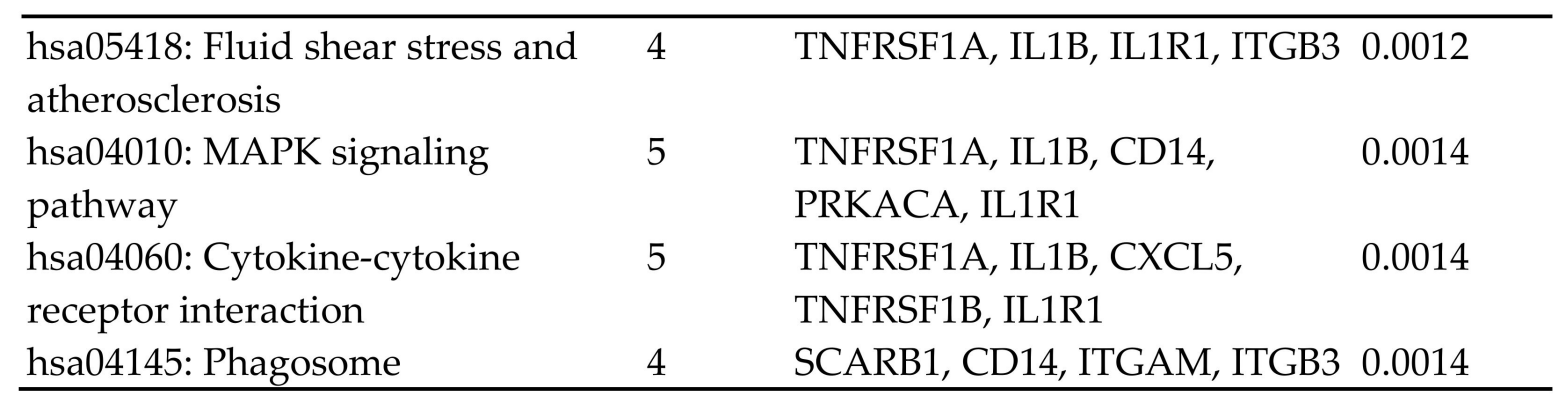
Interaction of proteins coded by differentially expressed genes in CAD patients. (**a**) the full STRING network was created with Cytoscape. The number of nodes is 28, number of edges is 110, and PPI enrichment is lower 1.0 × 10^−16^. Upregulated and downregulated genes are marked by red and blue colors, respectively. (**b**) Top 13 KEGG pathways were sorted by gene number (4–9) and FDR values lower than 0.0015.

The PPI network created with STRING and exported to Cytoscape is given in Figure 2a for protein products of 28 DEGs. Thirteen of the forty significant KEGG pathways sorted by FDR and gene count (equal or higher than 4) are included in Figure 2b. They included cholesterol metabolism, Hematopoietic cell lineage, Amoebiasis, PPAR signaling pathway, Pertussis, Human cytomegalovirus infection, NF-kappa B signaling pathway, TNF signaling pathway, Osteoclast differentiation, Fluid shear stress and atherosclerosis, MAPK signaling pathway, Cytokine–cytokine receptor interaction, and Phagosome. Furthermore, the Toll-like receptor signaling pathway and the IL-17 signaling pathway, each with three participating nodes, were detected as well. Thus, both lipid, inflammation, and innate immunity-associated pathways are significant in CAD.

### 3.3. Association of Transcripts with Lipids in Control and CAD Cohorts

#### 3.3.1. Bivariate Correlations between Transcript and Lipid Levels

The associations between lipid and apolipoprotein content and gene expression were studied by correlation analysis for two patient cohorts separately. The significant correlations for the HDL cluster given as Pearson coefficients are included in Table 3.

For the control cohort, HDL-C was negatively associated with cubilin (*CUBN*) and sterol O-acyltransferase 1 (*SOAT1*) transcripts. Total cholesterol was positively associated with *APOA1*, while it was negatively associated with *CUBN* and high-density lipoprotein binding protein (*HDLBP*). ApoA-I was negatively associated with the lecithin-cholesterol acyltransferase (*LCAT*) transcript.

For CAD patients, HDL-C was positively associated with the transcripts of genes coding cholesterol transporter ATP binding cassette subfamily G member 1 (*ABCG1*), albumin (*ALB*), and other proteins involved in cholesterol metabolism (*CUBN*, *HDLBP*, and protein kinase cAMP-activated catalytic subunit gamma (*PRKACG*)). However, HDL-C was negatively associated with bone morphogenetic protein 1 (*BMP1*), *LCAT*, *SOAT1,* and protein kinase cAMP-activated catalytic subunit beta (*PRKACB*) transcripts. Importantly, HDL-C was negatively associated with the 3-hydroxy-3-methylglutaryl-CoA reductase (*HMGCR*) transcript. The associations between apoA-I and transcript levels generally included the above-mentioned associations for HDL-C with the addition of a positive association between apoA-I and amnion-associated transmembrane protein (*AMN*) transcript and a negative association between apoA-I and phospholipid transfer protein (*PLTP*). Plasma TG were associated with transcripts generally in a manner reciprocal to HDL-C.

The significant associations for the transcripts of atherogenesis-prone genes are included in Table 4. In the control cohort, HDL-C was negatively associated with microsomal glutathione S-transferase 1 (*MGST1*) and *TLR8* transcripts. Total cholesterol and TG were not associated with any transcript in the atherogen cluster.

For CAD patients, HDL-C was positively associated with the transcripts of two genes: protein kinase C theta (*PRKCQ*) and sterol regulatory element binding transcription factor 1 (*SREBF1*). HDL-C and apoA-I were negatively associated with the following fourteen transcripts: CD14 molecule (*CD14*), CD36 molecule (*CD36*), cytochrome b-245 alpha chain (*CYBA*), coagulation factor V (*F5*), *MGST1*, NPC intracellular cholesterol transporter 2 (*NPC2*), oxidized low-density lipoprotein receptor 1 (*OLR1*), S100 calcium-binding protein A12 (*S100A12*), S100 calcium-binding protein A8 (*S100A8*), S100 calcium-binding protein A9 (*S100A9*), secretory leukocyte peptidase inhibitor (*SLP1*), Toll-like receptor 5 (*TLR5*), Toll-like receptor 8 (*TLR8*), and vascular endothelial growth factor A (*VEGFA*). Plasma TG was associated with transcripts generally in a manner reciprocal to HDL-C. Notably, total cholesterol was negatively associated with *CD36* and *NPC2* transcripts, analogously to HDL-C.

#### 3.3.2. Contribution of Transcript to HDL-C Level by Multiple Regression

To estimate the contribution of gene transcripts to HDL-C levels, multiple linear regression was used for two cohorts separately. To increase statistical power, only transcripts significantly correlated with HDL-C and passed through the control for multiple comparisons, plasma TG, non-HDL-C, and age were included in the analysis as independent variables. Multiple regression data are included in Table 5. For the control cohort, HDL-C was positively associated with non-HDL-C while negatively associated with *TLR8*, *SOAT1,* and plasma TG. Four independent predictors with VIF values close to 1 controlled up to 59% of HDL-C variability. For CAD patients, HDL-C was positively associated with *PRKACG*, *PRKCQ*, *SREBF1,* and nonHDL-C, while negatively associated with *PRKACB*, *LCAT*, *S100A8,* and plasma TG. Eight independent predictors with VIF values 1.18–1.68 controlled up to 79% of HDL-C variability.

## 4. Discussion

Numerous studies have sought to identify gene expression signatures of CAD risk by measuring mRNA in peripheral blood, but only a few candidate transcripts have been validated across studies [43]. The use of two gene sets related to HDL metabolism and atherogenesis predefined by us earlier by the system biology approach [31] permitted us to reach a 43% hit rate (28 from 65 genes) to reveal gene expression changes at CAD. Notably, two gene clusters were selected ab initio, thus eliminating the labor-consuming transcriptomics sequencing analysis in experimental studies [44,45] or the initial search for gene significance in in silico approaches [46,47].

The major novelties of our study are, firstly, the use of peripheral blood mononuclear cells (PBMC) from CAD patients unaffected by lipid-lowering therapy known to profoundly change lipoprotein metabolism; secondly, the down-regulation of the expression of genes involved in cholesterol efflux and reverse cholesterol transport in CAD; thirdly, the involvement of six signaling pathways connected to inflammation and innate immunity in CAD; and, finally, the different contribution of transcript level to HDL-C concentration for CAD and control patients. A gender-specific approach was applied in our study due to a higher CAD mortality rate in men compared to women, a different clinical picture of coronary heart disease, and distinctive risk factors in women [48]. Thus, the associations of HDL-C level with the expression of genes involved in HDL metabolism and atherogenesis for a future targeted personalized therapy are primarily limited to only middle-aged male patients with CAD. The other limitation of the study is the absence of data on the level of protein products of the genes studied. However, a close coincidence of mRNA expression and protein levels is known [42], at least for several genes described in the present study. The direct relationship between transcript level and protein activity is assumed hereinafter.

Three aspects are important in the analysis of differential gene expression at CAD and the contribution of transcripts to HDL-C level: (1) RCT and cholesterol efflux; (2) cholesterol synthesis and uptake; (3) intracellular cholesterol trafficking [49]. The data on the expression profile and contribution of transcript level to HDL-C concentration are discussed successively.

By differential expression, the expression of *NR1H2* and *NR1H3* LXR-receptors was up-regulated in CAD patients compared to controls, which may be related to the accumulation of intracellular cholesterol in coronary artery disease. Moreover, the expression of *ABCA1*, *ABCA5*, *SCARB1*, *ALB*, *APOA1*, and *LCAT*, all belonging to the major participants in reverse cholesterol transport, was down-regulated, evidencing RCT deficiency at CAD. Notably, *LDLR* and *APOE* were up-regulated; apoE may compensate for apoA-I deficiency in the cholesterol efflux reaction [50]. The increased expression of *LDLR* in CAD patients compared to controls seems to underlie the decrease of LDL-C in the plasma of CAD patients due to the increased clearance of LDL through the hepatic LDL receptor [51]. Based on the decreased cholesterol content in a single LDL particle, an increased percentage of cholesterol-depleted small dense LDL (sdLDL) in CAD patients is suggested. These particles demonstrate prolonged circulation time, likely due to a lower affinity for the LDL receptor [52]. The increased expression of *LPL* at CAD, which stimulates selective uptake of cholesteryl esters from LDL via pathways that are distinct from SR-BI [53], seems to also contribute to the generation of sdLDL. Furthermore, the LPL present in the arterial wall may, by binding to LDL, increase the residence time of LDL in the arterial wall, thus promoting atherogenesis [54]. However, the change in intracellular cholesterol levels is not definitely evident. A simplest sequential kinetic scheme of reverse cholesterol transport may include (i) cholesterol efflux to lipid-free apoA-I by ABCA1 in macrophages; (ii) cholesteryl ester formation in nascent preβ-HDL and mature α-HDL catalyzed by LCAT; (iii) selective removal of cholesteryl ester from α-HDL by SR-BI in hepatocytes with the generation of CE-depleted HDL*: apoA-I (via ABCA1) → preβ-HDL (via LCAT) → α-HDL → HDL* (via SR-BI). Within this scheme, cholesterol efflux is a first and rate-limiting step, and a residual RCT efficiency of 73–79% in CAD compared to control may be reasonably assumed based on *APOA1* and *ABCA1* expression changes (Figure 1).

The systemic inflammation in CAD patients is evidenced by several findings. First, the proinflammatory *IL1B* is up-regulated. Antagonism of interleukin–1β reduces coronary heart disease in patients with a previous myocardial infarction and evidence of systemic inflammation. IL-1β and IL-18 are produced via the NLRP3 inflammasome in myeloid cells in response to cholesterol accumulation [28], thus supporting our suggestion on the accumulation of intracellular cholesterol in PBMC from CAD patients. The up-regulation of *IL1B* and *TNFRSF1A* genes contributes to the NF-kappa B signaling pathway. Second, the increased expression of *TLR5* and *TLR8* as dominant components of the innate immune system [55] contributes to the Toll-like receptor signaling pathway. Third, *CXCL5* chemokine expression was up-regulated. CXCL5 has pro-inflammatory effects, mainly through the recruitment of monocytes by the CXCR2 receptor [56].

The above-mentioned proinflammatory shift in gene expression at CAD may be counterbalanced by down-regulation of *IL1R1*, *CD14*, *TNFRSF1B*, *S100A8*, and *ITGAM* genes. First, IL1R1/IL1β augment both megakaryocyte and platelet functions, thereby promoting a prothrombotic environment and potentially contributing to the development of atherothrombotic disease [57]. Second, CD14 is suggested to underlie the contribution of intermediate CD14++CD16+ monocyte counts that predict cardiovascular events [58]. Third, TNFRSF1B has been related to cardiovascular disease and mortality in the Framingham Heart Study [59]. Fourth, S100A8 has mainly been implicated in cardiovascular disease [60]. Finally, ITGAM has been identified as an inducer of coronary atherosclerosis through endothelial cell dysfunction [61]. Moreover, the expression of the adhesion receptor integrin *ITGB3* was up-regulated at CAD in the present study. The ITGB3 molecule is consistently detected on macrophages in early and advanced human atherosclerotic lesions, and its expression is up-regulated by atherogenic stimuli [62]. Furthermore, macrophage beta3 integrin, acting through TNFalpha, suppressed inflammation caused by hyperlipidemia attributable to high-fat feeding [63].

For the control cohort, the significance of transcript level as independent predictor of HDL-C variability by multiple regression revealed the negative contributions of *TLR8* and *SOAT1* expression to HDL-C variability. *TLR8,* as a negative predictor of HDL-C level and a dominant component of the innate immune system [55], assumes HDL’s atheroprotective property. *SOAT1* as a negative predictor of HDL-C level may be associated with the increase in intracellular free cholesterol pool available for efflux at hyperalphalipoproteinemia in controls. The negative contribution of plasma TG to HDL-C level may originate first from the generation of nascent HDL [64] at TG lipolysis in TG-rich lipoproteins and, second, from the increased hetero-exchange of cholesteryl ester and triglyceride molecules between HDL and VLDL particles at the increase of plasma TG with the subsequent hydrolysis of TG in HDL [3].

For the CAD cohort, eight independent predictors of HDL-C variability were revealed by multiple regression. A positive contribution of *SREBF1* expression up-regulated by liver X receptors assures a supply of fatty acids during sterol overloading at hyperalphalipoproteinemia to allow storage of excess cholesterol as cholesteryl ester [16]. The *LCAT* transcript as a negative predictor of HDL-C level assumes RCT impairment at CAD. *PRKACG* is a central actor in platelet biogenesis [65] and also a positive predictor of HDL-C level. The latter may be associated with the increase in platelet activation with the increase in HDL-C at CAD. The loss of the *PRKAC*β2 splice variant, the latter being measured in our study, can increase the inflammatory susceptibility of macrophages [66] and Cβ2 transcripts were reduced in human fibroatheroma, which may cause plaque progression [67]. Thus, HDL’s proatherogenic property follows from the negative contribution of *PRKACB* to HDL-C variability. Also, the proatherogenic property of HDL deducted from the positive contribution of *PRKCQ* to HDL-C variability follows from the involvement of protein kinase C theta in thrombin-induced CD36 expression and foam cell formation; the inhibition of protein kinase C theta decreases atherosclerotic lesions and improves insulin sensitivity [68]. *S100A8* has mainly been implicated in cardiovascular disease by promoting vascular endothelial cell dysfunction and apoptosis [60]. The negative contribution of *S100A8* to HDL-C levels thereby assumes HDL’s atheroprotective property in CAD patients. Overall, both atheroprotective (via *S100A8* expression) and proatherogenic (via *SREBF1*, *LCAT*, *PRKACG*, *PRKACB*, and *PRKCQ* expression) properties of HDL seem to exist in CAD patients. However, the causal relationships between HDL-C and transcript levels remain to be elucidated.

## 5. Conclusions

For control patients, the negative contributions of *TLR8* and *SOAT1* transcripts to HDL-C levels assume “full” HDL functionality. However, for CAD patients, the efficiency of reverse cholesterol transport is 73–79%, and intracellular cholesterol accumulates with the increase of HDL-C. More functional and compositional studies are needed to evaluate the “residual” HDL functionality. The measurements of the cholesterol efflux capacity of HDL preparations are now in progress. Also, the future estimations of the expression profiles for low, normal, and elevated HDL-C concentrations in CAD seem to clarify the existing uncertainty about the exact nature of HDL functionality. Systemic inflammation in CAD patients may be limited by up-regulation *of APOE* and *ITGB3* and down-regulation of *IL1R1*, *CD14*, *TNFRSF1B*, *S100A8*, and *ITGAM*. Both the selected key genes and signal pathways, derived from analysis of protein–protein interactions, may represent HDL-specific targets for diagnosis and treatment of atherogenesis.

## Figures and Tables

**Table 1:** Laboratory and anthropometric parameters for two cohorts of patients. Mean ± SD values and their comparison by Mann–Whitney test are included.

	Control (*n* = 63)	CAD (*n* = 76)	*p*
HDL-C, mM	1.25 ± 0.43	1.21 ± 0.57	0.133
Chol, mM	5.04 ±1.28	4.21 ± 1.32 ^&^	0.000
LDL-C, mM	3.08 ± 1.04	2.32 ± 0.97 ^&^	0.000
VLDL-C, mM	0.72 ± 0.34	0.68 ± 0.31	0.559
nonHDL-C, mM	3.79 ± 1.10	3.00 ± 1.04 ^&^	0.000
TG, mM	1.55 ± 0.74	1.48 ± 0.68	0.697
atherogenicity index	3.32 ± 1.25	2.87 ± 1.40 ^&^	0.017
apoA-I, mg/dL	140.51 ± 38.19	157.83 ± 48.7 ^&^	0.027
apoB, mg/dL	87.32 ± 20.67	84.27 ± 25.37	0.259
BMI, kg/m^2^	28.63 ± 2.75	28.14 ± 3.57	0.599
age, year	49.05± 5.63	54.07 ± 4.18 ^&^	0.000

^&^ significant difference between CAD and control cohorts (*p* < 0.05).

**Table 2:** Gene Ontology functional enrichment for DEGs in CAD relative to control. The FDR values are given.

Term	Count	Genes	FDR
**Biological Process**			
GO:0042632~cholesterol homeostasis	11	ABCA1, SCARB1, CETP, ABCA5, NR1H2, LPL, LCAT, NR1H3, APOA1, APOE, LDLR	5.03 × 10^−14^
GO:0034375~high-density lipoprotein particle remodeling	7	SCARB1, CETP, ABCA5, LCAT, APOA1, APOE, PLTP	8.94 × 10^−12^
GO:0043691~reverse cholesterol transport	7	ABCA1, SCARB1, CETP, ABCA5, LCAT, APOA1, APOE	1.38 × 10^−11^
GO:0010745~negative regulation of macrophage derived foam cell differentiation	6	ABCA1, CETP, ABCA5, NR1H2, ITGB3, NR1H3	6.34 × 10^−10^
GO:0006869~lipid transport	8	ABCA1, SCARB1, CETP, ABCA5, APOA1, PCTP, APOE, PLTP	4.64 × 10^−9^
GO:0010875~positive regulation of cholesterol efflux	6	ABCA1, NR1H2, NR1H3, APOA1, APOE, PLTP	2.62 × 10^−8^
GO:0034372~very-low-density lipoprotein particle remodeling	5	CETP, LPL, LCAT, APOA1, APOE	2.99 × 10^−8^
GO:0070328~triglyceride homeostasis	6	SCARB1, CETP, LPL, NR1H3, APOA1, APOE	8.82 × 10^−8^
GO:0008203~cholesterol metabolic process	7	ABCA1, CETP, ABCA5, LCAT, APOA1, APOE, LDLR	8.82 × 10^−8^
GO:0030301~cholesterol transport	5	CETP, ABCA5, LCAT, APOA1, LDLR	1.20 × 10^−6^
GO:0033344~cholesterol efflux	5	ABCA1, SCARB1, ABCA5, APOA1, APOE	2.61 × 10^−6^
GO:0006954~inflammatory response	9	IL1R1, IL1B, S100A12, TLR8, CD14, TNFRSF1B, CXCL5, S100A8, TNFRSF1A	2.95 × 10^−6^
GO:0034384~high-density lipoprotein particle clearance	4	SCARB1, APOA1, APOE, LDLR	3.62 × 10^−6^
GO:0015914~phospholipid transport	5	SCARB1, CETP, PCTP, PLTP, LDLR	9.50 × 10^−6^
GO:0050729~positive regulation of inflammatory response	6	IL1B, LPL, S100A12, LDLR, S100A8, TNFRSF1A	1.34 × 10^−5^
GO:0034380~high-density lipoprotein particle assembly	4	ABCA1, APOA1, APOE, PRKACA	1.84 × 10^−5^
GO:0010867~positive regulation of triglyceride biosynthetic process	4	SCARB1, NR1H2, NR1H3, LDLR	3.57 × 10^−5^
GO:0006898~receptor-mediated endocytosis	5	AMN, ITGAM, CD14, APOE, LDLR	1.23 × 10^−4^
GO:0071222~cellular response to lipopolysaccharide	6	ABCA1, IL1B, NR1H3, CD14, TNFRSF1B, CXCL5	1.51 × 10^−4^
GO:0006629~lipid metabolic process	6	NR1H2, LPL, LCAT, NR1H3, PLTP, LDLR	2.98 × 10^−4^
GO:0032369~negative regulation of lipid transport	3	NR1H2, ITGB3, NR1H3	2.98 × 10^−4^
GO:0042158~lipoprotein biosynthetic process	3	LCAT, APOA1, APOE	4.73 × 10^−4^
GO:0032376~positive regulation of cholesterol transport	3	CETP, NR1H2, NR1H3	6.50 × 10^−4^
GO:0070508~cholesterol import	3	SCARB1, APOA1, LDLR	6.50 × 10^−4^
GO:0032489~regulation of Cdc42 protein signal transduction	3	ABCA1, APOA1, APOE	0.001163
GO:0051006~positive regulation of lipoprotein lipase activity	3	NR1H2, NR1H3, APOA1	0.001794
GO:0010887~negative regulation of cholesterol storage	3	ABCA1, NR1H2, NR1H3	0.002109
GO:0033700~phospholipid efflux	3	ABCA1, APOA1, APOE	0.00288
GO:0071260~cellular response to mechanical stimulus	4	IL1B, ITGB3, TLR8, TNFRSF1A	0.003053
GO:0045723~positive regulation of fatty acid biosynthetic process	3	NR1H2, NR1H3, APOA1	0.003133
GO:0032729~positive regulation of interferon-gamma production	4	IL1R1, IL1B, TLR8, CD14	0.003204
GO:0030593~neutrophil chemotaxis	4	IL1B, S100A12, CXCL5, S100A8	0.003466
GO:0055091~phospholipid homeostasis	3	ABCA1, CETP, APOA1	0.004246
GO:0051044~positive regulation of membrane protein ectodomain proteolysis	3	IL1B, APOE, TNFRSF1B	0.004632
GO:0046470~phosphatidylcholine metabolic process	3	CETP, LCAT, APOA1	0.00616
GO:0050728~negative regulation of inflammatory response	4	NR1H3, APOA1, APOE, TNFRSF1A	0.012719
GO:0006641~triglyceride metabolic process	3	CETP, LPL, APOE	0.017257
GO:0031663~lipopolysaccharide-mediated signaling pathway	3	SCARB1, IL1B, CD14	0.022846
GO:0006644~phospholipid metabolic process	3	LPL, LCAT, APOA1	0.036972
GO:0090108~positive regulation of high-density lipoprotein particle assembly	2	ABCA1, NR1H2	0.036972
GO:0010899~regulation of phosphatidylcholine catabolic process	2	SCARB1, LDLR	0.036972
GO:0061771~response to caloric restriction	2	APOE, LDLR	0.036972
GO:0032757~positive regulation of interleukin-8 production	3	IL1B, TLR8, CD14	0.040866
GO:0002790~peptide secretion	2	ABCA1, S100A8	0.043243
GO:1902339~positive regulation of apoptotic process involved in morphogenesis	2	TNFRSF1B, TNFRSF1A	0.043243
GO:0090107~regulation of high-density lipoprotein particle assembly	2	ABCA1, LCAT	0.043243
**Molecular function**			
GO:0031210~phosphatidylcholine binding	5	ABCA1, CETP, APOA1, PCTP, PLTP	1.35 × 10^−5^
GO:0001540~beta-amyloid binding	5	SCARB1, ITGAM, APOA1, APOE, LDLR	3.40 × 10^−4^
GO:0008289~lipid binding	6	SCARB1, CETP, APOA1, PCTP, APOE, PLTP	3.40 × 10^−4^
GO:0034186~apolipoprotein A-I binding	3	ABCA1, SCARB1, LCAT	6.56 × 10^−4^
GO:0071813~lipoprotein particle binding	3	LPL, APOE, LDLR	0.001294
GO:0015485~cholesterol binding	4	ABCA1, CETP, NR1H3, APOA1	0.001294
GO:0008035~high-density lipoprotein particle binding	3	SCARB1, APOA1, PLTP	0.002458
GO:0030169~low-density lipoprotein particle binding	3	SCARB1, PLTP, LDLR	0.004408
GO:0034185~apolipoprotein binding	3	ABCA1, SCARB1, LPL	0.004408
GO:0005319~lipid transporter activity	3	ABCA1, ABCA5, APOE	0.011161
GO:0005102~receptor binding	5	ABCA1, AMN, LPL, APOA1, APOE	0.030793
GO:0005507~copper ion binding	3	ALB, S100A12, F5	0.035664
**Cellular component**			
GO:0005615~extracellular space	16	CETP, AMN, ITGAM, APOA1, LPL, LCAT, CXCL5, F5, TNFRSF1A, IL1B, ALB, S100A12, CD14, APOE, PLTP, S100A8	2.71 × 10^−7^
GO:0005576~extracellular region	16	CETP, IL1R1, APOA1, LPL, LCAT, TNFRSF1B, CXCL5, F5, TNFRSF1A, IL1B, ALB, S100A12, CD14, APOE, PLTP, S100A8	4.41 × 10^−7^
GO:0034364~high-density lipoprotein particle	5	CETP, LCAT, APOA1, APOE, PLTP	1.55 × 10^−6^
GO:0005886~plasma membrane	19	ABCA1, SCARB1, AMN, ITGAM, ABCA5, IL1R1, ITGB3, APOA1, LPL, TNFRSF1B, F5, TNFRSF1A, S100A12, TLR8, CD14, APOE, PRKACA, LDLR, S100A8	2.95 × 10^−4^
GO:0009897~external side of plasma membrane	7	ABCA1, ITGAM, IL1R1, ITGB3, TLR8, CD14, LDLR	6.99 × 10^−4^
GO:0070062~extracellular exosome	12	SCARB1, CETP, AMN, ITGAM, ITGB3, ALB, LCAT, APOA1, CD14, APOE, PRKACA, S100A8	0.001074
GO:0042627~chylomicron	3	LPL, APOA1, APOE	0.002452
GO:0043235~receptor complex	5	AMN, ITGB3, NR1H3, LDLR, TNFRSF1A	0.002594
GO:0034361~very-low-density lipoprotein particle	3	LPL, APOA1, APOE	0.004377
GO:0009986~cell surface	6	SCARB1, ITGAM, ITGB3, LPL, LDLR, TNFRSF1A	0.014727
GO:0005794~Golgi apparatus	7	ABCA1, ABCA5, ALB, CD14, APOE, LDLR, F5	0.031004
GO:0030139~endocytic vesicle	3	ABCA1, AMN, APOA1	0.033311
GO:0045121~membrane raft	4	ABCA1, CD14, TNFRSF1B, TNFRSF1A	0.033311
GO:0010008~endosome membrane	4	AMN, TLR8, CD14, LDLR	0.037424
GO:0002947~tumor necrosis factor receptor superfamily complex	2	TNFRSF1B, TNFRSF1A	0.032813
GO:0005788~endoplasmic reticulum lumen	4	ALB, APOA1, APOE, F5	0.041851
GO:0005764~lysosome	4	SCARB1, ABCA5, IL1B, LDLR	0.041851

**Table 3:** Correlations between expression of genes within the HDL cluster and plasma lipoprotein and apoA-I levels in control and CAD cohorts. Pearson correlation coefficients *r* and *p*-values in brackets are given only for significant correlations (*p* < 0.05) and controlled with the Benjamini–Hochberg procedure with FDR values of 0.20 (control) and 0.05 (CAD cohort).

Gene	HDL-C	TG	Chol	apoA-I
Control				
*APOA1*			0.323 (0.011)	
*CUBN*	−0.322 (0.010)		−0.305 (0.015)	
*HDLBP*			−0.324 (0.010)	
*LCAT*				−0.336 (0.008)
*SOAT1*	−0.397 (0.002)			
CAD				
*ABCG1*	0.294 (0.010)			0.273 (0.018)
*ALB*	0.451 (0.000)		0.343 (0.002)	0.453 (0.000)
*AMN*				0.304 (0.008)
*BMP1*	−0.332 (0.003)			−0.345 (0.002)
*CUBN*	0.546 (0.000)	−0.375 (0.001)		0.553 (0.000)
*HDLBP*	0.335 (0.003)			0.371 (0.001)
*HMGCR*	−0.314 (0.006)			−0.319 (0.005)
*LCAT*	−0.319 (0.005)		−0.330 (0.004)	−0.426 (0.000)
*PLTP*				−0.287 (0.012)
*PRKACB*	−0.438 (0.000)	0.366 (0.001)		−0.475 (0.000)
*PRKACG*	0.414 (0.000)	−0.345 (0.002)		0.394 (0.001)
*SOAT1*	−0.353 (0.002)			−0.388 (0.001)

**Table 4:** Correlations between expression of genes within the atherogen cluster and plasma lipoprotein and apoA-I levels in control and CAD cohorts. Pearson correlation coefficients (*r* and *p* values in brackets) are given only for significant correlations (*p* < 0.05) and controlled with the Benjamini–Hochberg procedure with an FDR value of 0.05.

Gene	HDL-C	TG	Chol	apoA-I
Control				
*MGST1*	−0.393 (0.002)			−0.434 (0.001)
*TLR8*	−0.378 (0.002)			
CAD				
*CD14*	−0.430 (0.000)			−0.426 (0.000)
*CD36*	−0.570 (0.000)	0.335 (0.003)	−0.441 (0.000)	−0.571 (0.000)
*CYBA*	−0.334 (0.003)			−0.363 (0.001)
*F5*	−0.357 (0.002)			−0.364 (0.001)
*MGST1*	−0.270 (0.018)			−0.333 (0.003)
*NPC2*	−0.357 (0.002)		−0.366 (0.001)	−0.370 (0.001)
*OLR1*	−0.367 (0.001)	0.416 (0.000)		−0.346 (0.002)
*PRKCQ*	0.455 (0.000)			0.429 (0.000)
*S100A12*	−0.438 (0.000)			−0.443 (0.000)
*S100A8*	−0.452 (0.000)			−0.413 (0.000)
*S100A9*	−0.406 (0.000)			−0.364 (0.001)
*SLPI*	−0.381 (0.001)	0.430 (0.000)		−0.396 (0.000)
*SREBF1*	0.316 (0.005)			
*TLR5*	−0.322 (0.005)			−0.354 (0.002)
*TLR8*	−0.349 (0.002)			−0.337 (0.003)
*VEGFA*	−0.346 (0.002)			−0.232 (0.004)

**Table 5:** The contribution of plasma lipid and transcripts of genes from HDL-cluster and atherogen cluster to HDL-C levels in control and CAD cohorts. Transcripts significantly correlated with HDL-C by pairwise comparison were included, together with age, TG, and non-HDL-C as independent variables. Forward and backward stepwise multiple linear regression analysis was performed until the coincidence of both equations. R^2^ value equals explained variability. The variance inflation factor (VIF) is a measure of multicollinearity (values lower than 4 correspond to negligible multicollinearity). The Darbin–Watson statistic DW serves as a *test* for checking autocorrelation in the residuals (no first-order autocorrelation is assumed for DW values 1.50–2.50).

	Independent Variable	β ± SEM	*p*	R^2^	VIF	DW
Control (*n* = 54)				0.594		1.76
	*SOAT1*	−0.521 ± 0.094	0.000		1.08	
	*TLR8*	−0.222 ± 0.095	0.023		1.08	
	nonHDL-C	0.265 ± 0.098	0.009		1.16	
	TG	−0.319 ± 0.098	0.002		1.16	
CAD (*n* = 75)				0.785		1.70
	*PRKACB*	−0.273 ± 0.064	0.000		1.27	
	*PRKACG*	0.190 ± 0.062	0.003		1.18	
	*LCAT*	−0.340 ± 0.071	0.000		1.53	
	*PRKCQ*	0.166 ± 0.074	0.028		1.68	
	*S100A8*	−0.216 ± 0.072	0.004		1.60	
	*SREBF1*	0.306 ± 0.071	0.000		1.54	
	nonHDL-C	0.206 ± 0.071	0.005		1.56	
	TG	−0.243 ± 0.074	0.002		1.70	

## Data Availability

The data presented in this study are available on request from the corresponding author.

## Supplementary Materials

The following supporting information can be downloaded at: https://www.mdpi.com/article/10.3390/cimb45080431/s1, Supplementary Table S1: The primers used in RT-PCR.
