# Supplementary material for: Differential Expression of Subsets of Genes Related to HDL Metabolism and Atherogenesis in the Peripheral Blood in Coronary Artery Disease
Source: Curr Issues Mol Biol. 2023 Aug 16;45(8):6823–41. doi: 10.3390/cimb45080431 (PMC10452992; doi:10.3390/cimb45080431)
Supplement: Supplementary file 1 [file cimb-45-00431-s001.zip › cimb-2558752-supplementary.pdf]

Table S1. The primers used in RT-PCR.

Primer	GeneBank	5'- 3' nucleotide sequence	location
		HDL-related genes	
ABCA1 (F)	NM_005502.3	ACAATGGAGCGG GGAAGAC	3205 - 3224
ABCA1 (R)		AAGCGGGCATAGAACCAGA	3385 - 3404
ABCA5 (F)	NM_018672.5	CAAAGGGATACCAGCCAACA	2967-2987
ABCA5 (R)		ACAGGGGTCCATTCCAGC	3145-3163
ABCG1 (F)	NM_004915.3	GGAGAAGGATGAAGGCAGAAG	667 - 688
ABCG1 (R)		AAGAACATGACTGGAGGGTTG	805 - 826
ALB (F)	NM_000477.6	GAGCAGCTTGGAGAGTACA	1347 - 1366
ALB (R)		GTTCAGGACCACGGATAGAT	1516 - 1536
AMN (F)	NM_030943.3	CTCGCACCTGGACTGTG	306 - 323
AMN (R)		GCACTAGGCGGAAAGAAGA	458 - 477
APOA1 (F)	NM_000039.2	GTGTACGTGGATGTGCTCAA	487 - 507
APOA1 (R)		CACGCTGTCCCAGTTGTC	580 - 598
APOE (F)	NM_001302688.1	AGACCGAGTGGCAGAGCG	302 - 320
APOE (R)		GTCAGTTGTTCCTCCAGTTCCG	479 - 500
A2M (F)	NM_000014.5	CAGGACAAGAGGAAGGAAGT	3858 - 3878
A2M (R)		AAGCGAGGAGCACATAGG	4009 - 4027
BMP1 (F)	NM_001199.3	CAATGGCTACTCTGCTCAC	1283 - 1302
BMP1 (R)		ATCTCGGACCTCCACATAG	1400 - 1419
CETP (F)	NM_000078.2	CGTCATCACAGCCTCCTACCT	771 - 790
CETP (R)		AACTCGTCTCCCATCAGGCT	964 - 983
CUBN (F)	NM_001081.3	GGCAGATACTGTGGAAACAC	7274 - 7294
CUBN (R)		GGATTTGGGTTCGGGTAGTT	7451 - 7451
HDLBP (F)	NM_005336.5	CGCCAAGCCAGAATACCACA	2574 - 2593
HDLBP (R)		AGTCTTCCACCACATTATCCAGG	2763 - 2785
HMGCR (F)	NM_000859.3	TTCCAGAGCAAGCACATTAGC	506-527
HMGCR (R)		GCAGCCAAAGCAGCACATA	680-699
LCAT (F)	NM_000229.1	CCTTCTGGCTCCTCAATGT	82 - 101
LCAT (R)		CGGTAGCACATCCAGTTCA	220 - 239
LDLR (F)	NM_000527.4	GAGGTGGCCAGCAATAGAA	1490 - 1509
LDLR (R)		GATGACGGTGTCATAGGAAGAG	1572 - 1594
LIPC (F)	NM_000236.2	CTTCAACTCCTCCCTGCCTCT	285 - 305
LIPC (R)		TGGTGTAGTGGTCGTGGGC	433 - 451

LPL (F)	NM_000237.2	ACTGGCTGTCACGGGCTCA	705 – 723
LPL (R)		GGCTCCAAGGCTGTATCCCAA	836 – 856
PLTP (F)	NM_006227.3	GCAGGAGGAAGAGCGGAT	1068 – 1087
PLTP (R)		ACAATGCTCCCAAAGTAGGTG	1212 – 1232
PRKACA (F)	NM_002730.3	GTTCTCACACCTACGGCGGA	587 - 607
PRKACA (R)		GGCGAAACCGAAGTCTGTCA	748 - 768
PRKACB (F)	NM_182948.3	GCTATCCCCCATTCTTTGC	959 - 978
PRKACB (R)		CAATCTGTCGTGGCAAACC	1145 - 1164
PRKACG (F)	NM_002732.3	GGCGACATCAAGAACCACA	899 - 917
PRKACG (R)		GCTCTTCCTCCTCGTAGTCGT	1017 - 1103
SCARB1 (F)	NM_005505.4	CATCTACCCACCCAACGAAG	1189 - 1209
SCARB1 (R)		GAAGTGAGGATGGGAGAGAAAC	1273 - 1295
SOAT1 (F)	NM_003101.6	GCTGAGATGTTACGCTTTGGTG	1325-1346
SOAT1 (R)		ACGAAGAGCACGGGATAGAA	1559-1578
ZDHHC8 (F)	NM_001185024.1	CTCCAGCACCCTCTTCTTCGT	185 - 205
ZDHHC8 (R)		TCGTCCTCCTTGTCCTCATCC	341 - 361
Genes involved in atherogenesis			
ASGR2 (F)	NM_001181.4	GGAGAAACAGCAGCAGGACC	663 - 682
ASGR2 (R)		GAGTGAGAGAACCAGTAGCAGC	827 - 848
CD14 (F)	NM_000591.3	CAAGTGTGAAGCCTGGAAGC	277 - 296
CD14 (R)		ACAAGGTTCTGGCGTGGTC	436 - 454
CD36 (F)	NM_000072.3	CCTTTGCCTCTCCAGTTGAA	1310 - 1329
CD36 (R)		GTACACAGGTCTCCCTTCTTTG	1413 - 1434
CSF1R (F)	NM_005211.3	GGTGGCAGGAAGGTGATGT	836 - 854
CSF1R (R)		GGTGTTGTTGTGTTGGAGGA	999 - 1018
CSF2RB (F)	NM_000395.2	ATCCTCCTCTCCAACACCTCC	776 - 796
CSF2RB (R)		ACCTCCTTCCTCACCTCCCA	1001 - 1020
CXCL5 (F)	NM_002994.4	GCTGTTGGTGCTGCTGCT	208 - 225
CXCL5 (R)		CCGTTCTTCAGGGAGGCTAC	389 - 408
CYBA (F)	NM_000101.3	TTGTGTGCCTGCTGGAGTA	214 - 232
CYBA (R)		AGTAGGTAGATGCCGCTCG	421 - 439
F5 (F)	NM_000130.4	CTGGCTGGTGGCTCCTAA	5781 - 5798
F5 (R)		ATCTTGCTAATCTGGGCTCC	5941 - 5960
JAM3 (F)	NM_032801.4	TTCCAGAGCCAATCCCAGA	708 - 726
JAM3 (R)		TCCGCCAATGTTCAGGTC	874 - 891
ICAM1 (F)	NM_000201.2	GTGACCGTGAATGTGCTCTCC	1727 - 1747

ICAM1 (R)		GAGGCGTGGCTTGTGTGTT	1895 - 1913
IL1B (F)	NM_000576.2	TGTCCTGCGTGTTGAAAGATGA	641 - 661
IL1B (R)		CTGCTTGAGAGGTGCTGATGTA	796 - 856
IL1R1 (F)	NM_000877.3	TGCTTACTGGAAGTGGAATG	1099 - 1118
IL1R1 (R)		TGCTGCATCTATACCATGTG	1278 - 1297
IL18 (F)	NM_001562.3	GACCAAGGAAATCGGCCT	395 - 412
IL18 (R)		CACAGAGATAGTTACAGCCATACC	503 - 526
IL18RAP (F)	NM_003853.3	ACAACCCAGTCCGTCCAAC	1524 - 1542
IL18RAP (R)		ACATCAGGAAATAGGCTCAGG	1790 - 1810
IL18R1 (F)	NM_003855.3	CGATAAAGAAGAACGCCGAGT	655 - 675
IL18R1 (R)		GCAGAGCAGTTGAGCCTTACG	842 - 862
ITGAM (F)	NM_001145808.1	CTCTCTCCCAGGCTCCAGT	1827 - 1845
ITGAM (R)		CATTCCTTGCCACTTCCCT	1986 - 2004
ITGA2B (F)	NM_000419.4	AAGATTGTGCTGCTGGACG	2421 - 2439
ITGA2B (R)		GAAGGTGGATGCTGAGGTGA	2612 - 2631
ITGB3 (F)	NM_000212.2	AGTAACCTGCGGATTGGCTT	537 - 556
ITGB3 (R)		CACACTCTGCTTCTTCACTTCC	713 - 734
LTA (F)	NM_001159740.2	GCTGCTGCTGGTTCTGCT	282 - 299
LTA (R)		GTTCTGCTTGCTGGGGTCT	420 - 438
MGST1 (F)	NM_145792.2	CAGGTAATGGATGATGAAGTA	81 - 101
MGST1 (R)		GCCAAATGCTACACAGTCTTCT	206 - 227
MMP9 (F)	NM_004994.2	ACCCTTGTGCTCTTCCCTG	98 - 116
MMP9 (R)		CGACTCTCCACGCATCTCTG	192 - 211
NPC1 (F)	NM_000271.4	CAGCCACATAACCAGAGCGT	3778 - 3797
NPC1 (R)		AGCCAACACCACAATCCCT	3898 - 3916
NPC2 (F)	NM_006432.3	TCCCATTCCTGAGCCTGAT	362 - 380
NPC2 (R)		GTTGCCACTCCACCACCA	478 - 495
NR1H2 (F)	NM_007121.5	GCCATCATCTCAGTCCAGG	1104 - 1122
NR1H2 (R)		ACTCTGTCTCGTGGTTGTAGC	1237 - 1257
NR1H3 (F)	NM_005693.3	GCCTTGCTCATTGCTATCAG	1319 - 1338
NR1H3 (R)		GTGGGAACATCAGTCGGTCA	1447 - 1466
OLR1 (F)	NM_002543.3	TTGCCTGGGATTAGTAGTGACC	249 - 270
OLR1 (R)		CTTCTTCTGCTTGTTGCCG	376 - 394
PCTP (F)	NM_021213.3	GGTGAAGCAATACAAGCAGAG	578 - 598
PCTP (R)		TAATGAGCCAGGACGGAAT	672 - 690

PLA2G7 (F)	NM_005084.3	GGCATTGACCTGGCATCTC	759 - 777
PLA2G7 (R)		TGTGTCTCCTCCTCTTGTTTCAG	894 - 916
PRKCQ (F)	NM_006257.4	ACTGCCACCTTCTTCCCAC	587 - 605
PRKCQ (R)		CTTGAGTCCTTGCCGTGC	866 - 883
SLC7A11 (F)	NM_014331.3	GTCCGCAAGCACACTCCT	1358 - 1375
SLC7A11 (R)		ATGACGAAGCCAATCCCTG	1629 - 1647
SLPI (F)	NM_003064.3	CCTTCCTGGTGCTGCTTG	44 - 61
SLPI (R)		GACAACATCTCTTCTTCCCTGG	193 - 214
SOD2 (F)	NM_000636.3	CACCACAGCAAGCACCAC	309 - 326
SOD2 (R)		GTTCTCCACCACCGTTAGG	480 - 498
SREBF1 (F)	NM_001005291.2	CCTCAGATACCACCAGCGTC	1828 - 1847
SREBF1 (R)		TTGCGATGCCTCCAGAAGT	2026 - 2044
S100A8 (F)	NM_001319196.1	ATGCCGTCTACAGGGATGA	267 - 285
S100A8 (R)		ACGCCCATCTTTATCACCAG	407 - 426
S100A9 (F)	NM_002965.3	GACCATCATCAACACCTTCCAC	82 - 103
S100A9 (R)		TAGCCTCGCCATCAGCAT	284 - 301
S100A12 (F)	NM_005621.1	ATTAGGCTGGGAAGATGACAA	55 - 75
S100A12 (R)		GTGGGTGTGGTAATGGGCA	320 - 338
TLR5 (F)	NM_003268.5	GACCCTCTGCCCCTAGAATAA	581 - 601
TLR5 (R)		GCCATCAAAGGAGCAGGAA	707 - 725
TLR8 (F)	NM_016610.3	GGAACATCAGCAAGACCCATC	115 - 135
TLR8 (R)		CGCATAACTCACAGGAACCAGA	279 - 300
TNFRSF1A (F)	NM_001065.3	GCTCCTTCACCGCTTCAGA	563 - 581
TNFRSF1A (R)		GGTCCCATTGAGGCAGAGG	744 - 762
TNFRSF1B (F)	NM_001066.2	AACACACGCAGCCAACTCC	763 - 781
TNFRSF1B (R)		GTCACACCCACAATCAGTCCA	875 - 895
VEGFA (F)	NM_001025366.2	GAGGGCAGAATCATCACGAA	1136 - 1155
VEGFA (R)		CATCAGGGGCACACAGGA	1264 - 1281
Housekeeping genes			
GAPDH (F)	NM_002046.7	ATCATCCCTGCCTCTACTGG	692 - 711
GAPDH (R)		GAGTGGGTGTCGCTGTTGA	933 - 951
RPL3 (F)	NM_000967.3	GCATCGTGGGAAGGTGAAGA	140 – 159
RPL3 (R)		CCACAATGCCCACAACCAC	321 – 339
LDHA (F)	NM_005566.4	ATTCAGCCCGATTCCGTTAC	595 – 615
LDHA (R)		CCAATAGCCCAGGATGTGTAG	836 – 856
